# Toward Adversarial Robustness Network Intrusion Detection Based on Multi-Model Ensemble Approach

**DOI:** 10.3390/s26082478

**Published:** 2026-04-17

**Authors:** Thi-Thu-Huong Le, Jaehan Cho, Dawit Shin, Howon Kim

**Affiliations:** 1 Blockchain Platform Research Center, Pusan National University, Busan 46241, Republic of Korea; 2 School of Computer Science and Engineering, Pusan National University, Busan 46241, Republic of Korea; jhcho0208@gmail.com (J.C.); sin002277@gmail.com (D.S.)

**Keywords:** adversarial attacks, adversarial robustness, attack success rate (ASR), defense mechanisms, gradient-based attacks, intrusion detection systems (IDSs), network security

## Abstract

Machine learning-based network intrusion detection systems (NIDSs) remain vulnerable to adversarial manipulation, but the robustness literature for tabular NIDS data is still dominated by single-model, single-dataset, and non-adaptive evaluations. In this paper, we reposition the manuscript as a comparative robustness study of a four-component defense pipeline rather than as a claim of a universal defense primitive. We evaluate XGBoost, LightGBM, TabNet, and Residual MLP on RT_IOT2022 and Web_IDS23 under standard attacks, representative constrained/adaptive attacks, component-wise ablations, sample-fraction sensitivity, repeated-run significance tests, per-class F1 analysis, and computational-overhead measurements. The results show strong dataset and architecture dependence. On RT_IOT2022, tree-based models close most of the robustness gap under strong attacks but often only after large clean-accuracy reductions; Residual MLP achieves a more favorable balance, while the full defense stack over-regularizes TabNet. On Web_IDS23, aggregate robustness-gap reduction remains positive, yet simpler baselines such as adversarial-training-only or ensemble-only configurations frequently outperform the full four-stage pipeline in absolute clean/attack accuracy. Across both datasets, median filtering is the most fragile component: larger filter windows substantially degrade both clean and attacked accuracy, whereas contamination rate, anomaly-mixing weight, and ensemble size are comparatively stable. Representative constrained/adaptive evaluations reduce performance only modestly relative to standard FGSM/PGD, but per-class and overhead analyses show that minority-class collapse and training cost remain important deployment limitations. These findings support a more cautious conclusion: adversarial defense for tabular NIDS is validation driven and dataset specific, and the full defense stack should not be treated as a universal default.

## 1. Introduction

Machine learning-based network intrusion detection systems (NIDSs) are attractive because they can generalize beyond static signatures and recognize complex attack patterns in high-dimensional network-flow data [1,2]. At the same time, these systems are vulnerable to adversarial perturbations that alter packet-, flow-, or application-level features just enough to induce misclassification while preserving malicious intent [3,4,5]. For security applications, this robustness problem is especially important because even moderate drops in detection quality can translate into a substantial increase in undetected attacks.

A second challenge is methodological. Tabular NIDS robustness is harder to evaluate than image robustness because the feature space mixes protocol counters, timing statistics, and application-derived fields with very different semantics. As a result, some perturbations are best interpreted as generic stress tests for model sensitivity, while others are closer to semantics-aware evasion attempts. Existing papers frequently conflate these roles, and they also tend to evaluate only one model family or one dataset, making it difficult to judge whether an observed defense benefit is architectural, dataset specific, or merely an artifact of the evaluation protocol [6,7].

The present paper therefore reframes the paper as a comparative robustness evaluation of a four-component defense pipeline for tabular NIDS data. The pipeline combines input sanitization, ensemble diversity, adversarial training, and anomaly-aware calibration. Our goal is not to claim that this combination is a new universal defense primitive. Instead, we ask a more practical question: how do these components interact across different tabular model families and across two benchmark environments, and when does the full stack help, fail, or become unnecessarily expensive?

To answer that question, the paper broadens the empirical protocol in several ways. Beyond the original clean/attack accuracy analysis, we now add representative constrained/adaptive attacks, component-wise ablations against simpler baselines, parameter-sensitivity studies, sample-fraction analysis, per-class F1 heatmaps, repeated-run statistical significance comparisons, and explicit computational-overhead reporting. We also soften our deployment claims: the results are benchmark limited and should be interpreted as validation-driven evidence rather than proof of universal operational readiness.

We perform the comparative study across four model architectures that represent distinct tabular-learning paradigms: XGBoost [8], LightGBM [9], TabNet [10], and Residual MLP [11]. The study uses two benchmark datasets with different feature structures and attack surfaces: RT_IOT2022 [12], which emphasizes IoT traffic, and Web_IDS23 [13], which emphasizes web-application intrusion scenarios. The contrast between these two datasets is central to the paper because it reveals that robustness claims based on one environment do not automatically transfer to the other.

The main contributions of the study are as follows:

- We reposition the paper as a comparative robustness study for tabular NIDS models and explicitly separate universal-defense claims from dataset-specific empirical findings.
- We strengthen the evaluation with representative constrained/adaptive attacks, simpler ablation baselines, parameter-sensitivity analysis, sample-fraction analysis, per-class F1 results, repeated-run significance testing, and computational-overhead reporting.
- We show that the full four-component defense is not a universal winner: adversarial-training-only or ensemble-only baselines often provide better clean/robustness trade-offs, while median filtering is the most brittle component in the stack.
- We provide deployment-oriented guidance by identifying when robustness-gap reduction is meaningful, when it is merely a by-product of clean-accuracy collapse, and why dataset-specific validation remains necessary before operational adoption.

The remainder of the paper is organized as follows. Section 2 reviews prior work on adversarial attacks, defenses, and robustness evaluation for IDS. Section 3 describes the defense pipeline and the threat-model separation. Section 4 summarizes the experimental protocol, datasets, and hyperparameters. Section 5 presents the empirical results, and Section 6 synthesizes their implications for robust NIDS deployment. Section 7 concludes the paper.

## 2. Related Work

The vulnerability of machine learning-based intrusion detection systems to adversarial manipulation has emerged as a critical research area at the intersection of cybersecurity and adversarial machine learning [14,15,16]. This section reviews the existing work on adversarial attacks against IDS, defense mechanisms, robustness evaluation methodologies, and domain-specific applications.

Adversarial Attacks on Network Intrusion Detection. Early work by Biggio et al. [3] demonstrated that machine learning classifiers are vulnerable to evasion attacks at test time, establishing the foundation for adversarial ML in security applications. Corona et al. [5] extended this to intrusion detection, showing that adversaries can craft network traffic that evades detection while maintaining attack functionality. Subsequent research has explored various attack strategies, including gradient-based methods [4,17], and transferability attacks [18].

Recent studies have revealed the severity of adversarial threats to modern IDSs. Lin et al. [19] proposed IDSGAN, demonstrating that generative adversarial networks can craft realistic adversarial network flows that evade deep learning-based IDSs with high success rates. Zhang et al. [20] showed that even state-of-the-art deep learning IDS architectures suffer accuracy degradation exceeding 40% under FGSM and PGD attacks. Ahmed et al. [21] introduced constraint-aware adversarial attacks that respect protocol semantics and validity constraints, revealing that realistic perturbations can still significantly compromise IDS performance. Vitorino et al. [22] conducted a comprehensive robustness analysis using semantic perturbations that maintain network traffic validity, demonstrating that adversarial vulnerability persists even when attackers are constrained to realistic modifications.

Adversarial Defense Mechanisms for IDS. Defense strategies have evolved from simple adversarial training to sophisticated adaptive frameworks. Madry et al. [17] established adversarial training as a foundational defense, training models on adversarially perturbed examples to improve robustness. Tramèr et al. [23] proposed ensemble adversarial training, combining multiple models to increase attack difficulty. However, these general-purpose defenses often exhibit limited effectiveness when directly applied to network intrusion detection due to the unique characteristics of tabular network data.

Domain-specific defenses tailored for IDS have gained prominence. Xiong et al. [24] introduced AIDTF, an adversarial training framework specifically designed for network intrusion detection that integrates attacker and defender models in a black-box context, achieving improved robustness against targeted attacks. Chen et al. [25] proposed DYNAMITE, a dynamic defense selection framework that adaptively chooses defense strategies based on detected attack profiles, significantly reducing computational overhead while improving detection performance. Unlike DYNAMITE, which selects defenses based on detected attack profiles at inference time, our framework applies a static multi-component defense trained across diverse attack types, avoiding the overhead of runtime defense switching but potentially sacrificing adaptability. Building on adaptive approaches, Chen et al. [26] developed SAGE, a sample-aware guarding method leveraging active learning to improve defense stability and reduce performance gaps compared to static or oracle baselines. SAGE uses active learning to improve defense stability across datasets, whereas our approach reveals that even comprehensive ensemble defenses remain dataset sensitive, suggesting active learning alone may be insufficient without cross-dataset validation.

Input transformation defenses have also been explored. Xu et al. [27] proposed feature squeezing for detecting adversarial examples through dimensionality reduction and bit-depth reduction. Ennaji et al. [28] introduced behavior-aware defense methods for black-box adversaries, implementing lightweight context-aware perturbation techniques that confuse attackers without degrading IDS detection capability.

Model-Specific Robustness Analysis. Different model architectures exhibit distinct vulnerabilities to adversarial attacks [29]. Tree-based ensemble methods like XGBoost and LightGBM have shown both strengths and weaknesses. Chen et al. [30] demonstrated that decision trees can be robust against certain gradient-based attacks due to non-differentiable decision boundaries but remain vulnerable to optimization-based attacks. Recent work by Kantchelian et al. [31] revealed that gradient boosting models can be evaded through carefully designed mimicry attacks.

Deep learning architectures for tabular data have received increasing attention. TabNet [10,32], which employ attention mechanisms for feature selection, has shown promise for intrusion detection [33], but its adversarial robustness remains underexplored. Residual networks for tabular data [11] demonstrate strong baseline performance, yet their vulnerability to adversarial attacks in IDS contexts has not been systematically evaluated.

Domain-Specific Applications and Evaluations. Adversarial robustness research has extended to specialized network environments. Baldini et al. [34] investigated IDS robustness in 5G networks, combining extremely randomized trees with advanced feature selection to improve resilience against adversarial attacks in next-generation mobile infrastructure. Aloraini et al. [35] examined adversarial threats in vehicular networks, demonstrating that adversarial perturbations can reduce IDS accuracy by over 30% in connected and autonomous vehicle settings, emphasizing the need for domain-aware defenses. Hu et al. [36] applied reinforcement learning to guide adversarial training for IoT intrusion detection, achieving enhanced robustness against IoT-specific attack patterns. IoT environments present unique challenges due to resource constraints and heterogeneous device characteristics. Ferrag et al. [2] surveyed deep learning approaches for IoT security, highlighting adversarial vulnerabilities as a critical concern.

Robustness Evaluation Methodologies. Comprehensive robustness evaluation requires diverse metrics beyond simple accuracy. Carlini et al. [37] emphasized the importance of evaluating defenses against adaptive attacks and using multiple threat models. Athalye et al. [38] demonstrated that many proposed defenses fail against adaptive attackers who tailor attacks to defense mechanisms, highlighting the need for rigorous evaluation. Recent work advocates for evaluating ASR [39], confidence calibration [40], and robustness gap metrics [41,42] to capture different dimensions of adversarial robustness.

Cross-dataset evaluation has emerged as critical for assessing defense generalization. Sommer and Paxson [43] identified the “base-rate fallacy" and dataset bias as fundamental challenges in IDS evaluation. Apruzzese et al. [44] argued that many IDS evaluations fail to reflect real-world conditions due to dataset selection and preprocessing choices. Recent studies have begun examining cross-dataset transfer learning [45], though adversarial robustness under distribution shift remains largely unexplored.

Ensemble Learning and Multi-Model Defenses. Ensemble methods have shown promise for improving adversarial robustness. Pang et al. [46] demonstrated that ensemble diversity-training models with different architectures, loss functions, or data augmentation improve robustness beyond simple majority voting. Strauss et al. [47] proposed ensemble methods specifically for network intrusion detection, though without adversarial robustness analysis. Recent work by Yang et al. [48] introduced diversity regularization to explicitly maximize ensemble disagreement on adversarial examples.

Multi-model frameworks combining tree-based and neural approaches remain under explored. While hybrid architectures have been proposed for IDS [49], their adversarial robustness properties have not been systematically characterized. The interaction between different model types in ensemble defenses, particularly whether complementary vulnerabilities can be exploited to achieve superior robustness, represents an open research question.

Research Gaps and Positioning. Despite substantial progress, several critical gaps remain in adversarial robustness research for intrusion detection:

- Limited Cross-Dataset Evaluation: Most studies evaluate defenses on single datasets, making cross-environment generalization unclear. The phenomenon of negative defense transfer, where defenses degrade performance on out-of-distribution data, has received minimal attention.
- Incomplete Multi-Architecture Comparisons: Systematic comparisons of tree-based models (XGBoost and LightGBM) and modern neural architectures (TabNet and Residual MLP) under diverse adversarial attacks are lacking, preventing informed model selection for robust IDS deployment.
- Narrow Attack Portfolios: Many studies focus exclusively on gradient-based attacks (FGSM and PGD), neglecting noise-based and feature-level perturbations that may be more realistic in network environments with protocol constraints.
- Single-Metric Evaluation: Relying solely on accuracy metrics overlooks important robustness dimensions, including ASR, confidence calibration, and robustness–accuracy trade-offs that are critical for operational deployment.
- Lack of Unified Defense Frameworks: Existing defenses are often architecture specific or attack specific, limiting applicability across diverse IDS configurations. Comprehensive frameworks integrating complementary defense mechanisms are rare.

## 3. Methodology

This section presents our proposed adversarial-robust network intrusion detection framework based on multi-model ensemble learning. We first formulate the problem mathematically, then describe the overall framework architecture, followed by detailed explanations of each component, including adversarial attack generation, defense mechanisms, and ensemble prediction strategies.

### 3.1. Problem Formulation

Let $D={\left\{\left(x_{i},y_{i}\right)\right\}}_{i=1}^{N}$ denote the training dataset for network intrusion detection, where $x_{i}\in R^{d}$ represents a *d*-dimensional feature vector extracted from network traffic, and $y_{i}\in \left\{1,2,\ldots ,C\right\}$ denotes the corresponding attack class label from *C* possible categories. Our objective is to learn a robust classifier $f:R^{d}\to \left\{1,2,\ldots ,C\right\}$ that maintains high accuracy on both clean data and adversarial perturbed samples.

Clean Classification. For standard intrusion detection, we aim to minimize the expected risk:

(1) $$R\left(f\right)=E_{(x,y)∼D}\left[\ell (f(x),y)\right]$$

where ℓ(·,·) is a loss function, typically cross-entropy for multi-class classification:

(2) $$\ell \left(f\left(x\right),y\right)=-\log p_{y}\left(x\right)=-\log\frac{\exp(z_{y})}{\sum_{j=1}^{C}\exp\left(z_{j}\right)}$$

where z=f(x) represents the logits output by the model.

Adversarial Perturbation. An adversary seeks to craft perturbations $\delta \in R^{d}$ that cause misclassification while remaining imperceptible. Formally, an adversarial example $x_{adv}$ is generated by

(3) $$x_{adv}=x+\delta ,\;\mathrm{subject}\;\mathrm{to}\;{∥\delta ∥}_{p}\leq \epsilon$$

where ϵ is the perturbation budget and ${∥\cdot ∥}_{p}$ denotes the $\ell_{p}$ norm (typically p∈{2,∞}). The adversary’s objective is

(4) $$\max_{{∥\delta ∥}_{p}\leq \epsilon }\ell \left(f\left(x+\delta \right),y\right)$$

Adversarial Robustness. A robust classifier should minimize the worst-case risk under adversarial perturbations:

(5) $$R_{adv}\left(f\right)=E_{(x,y)∼D}\left[\max_{{∥\delta ∥}_{p}\leq \epsilon }\ell \left(f\left(x+\delta \right),y\right)\right]$$

Our proposed framework addresses this challenge through a combination of ensemble learning, adversarial training, and anomaly detection, aiming to achieve

(6) $$\min_{f\in F}\left[\left(1-\lambda \right)R\left(f\right)+\lambda R_{adv}\left(f\right)\right]$$

where F is the hypothesis space and λ∈[0,1] balances clean accuracy and adversarial robustness.

### 3.2. Framework Overview

**Figure 1:**
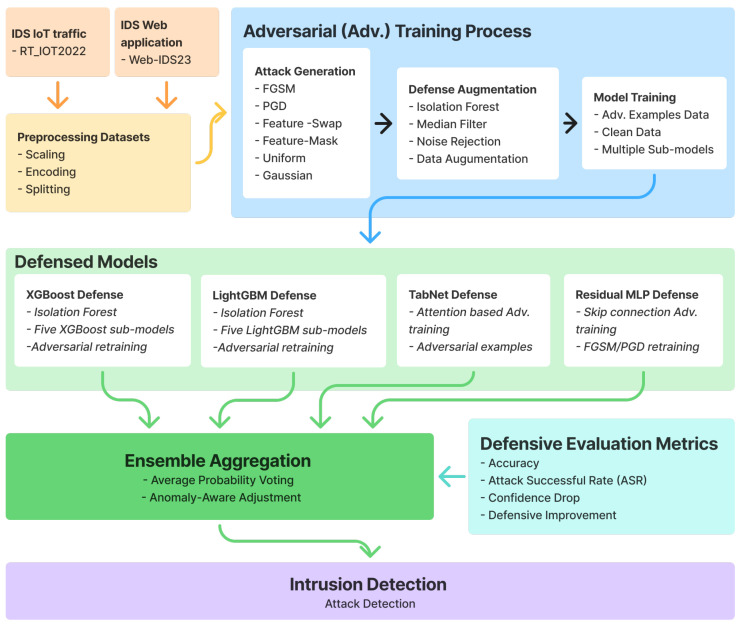
Multi-model adversarial defense framework for network intrusion detection.

Figure 1 illustrates our multi-model adversarial defense framework, which consists of four key components: (1) adversarial attack generation, (2) defense augmentation mechanisms, (3) defended model ensemble, and (4) anomaly-aware prediction. The framework operates in two phases: a training phase where models are hardened against adversarial examples, and an inference phase where ensemble predictions are made with anomaly detection.

We separate the evaluation into two threat-model families. The first family contains standard stress tests (Gaussian, Uniform, Feature-Mask, and Feature-Swap), used to probe generic sensitivity of tabular NIDS models. The second family contains gradient-based and defense-aware evaluations, used to test robustness under stronger attackers. We do not claim that every stress-test perturbation is semantics-preserving network evasion; rather, these attacks are reported as complementary robustness probes alongside stronger constrained/adaptive experiments.

#### 3.2.1. Gradient-Based Attacks

For differentiable neural models, we employ gradient-based methods that exploit the model’s loss surface directly. For tree-based models, FGSM/PGD-style evaluations are interpreted through transfer-based or score-based surrogates rather than true white-box gradients.

Fast Gradient Sign Method (FGSM). FGSM [4] generates adversarial examples through a single-step perturbation in the direction of the loss gradient:

(7) $$x_{adv}=x+\epsilon \cdot \mathrm{sign}\left(\nabla_{x}\ell \left(f\left(x\right),y\right)\right)$$

where sign(·) returns the element-wise sign of the gradient. The detailed FGSM attack procedure is presented in Algorithm 1.

**Algorithm 1** Fast Gradient Sign Method (FGSM) attack.
**Require:** Model *f*, input $x\in R^{d}$, true label *y*, perturbation budget ϵ
**Ensure:** Adversarial example $x_{adv}$

1:▹ Compute loss gradient2: L←CrossEntropy(f(x),y)3: $\nabla_{x}L\leftarrow \frac{\partial L}{\partial x}$
4:▹ Generate adversarial perturbation5: $\delta \leftarrow \epsilon \cdot \mathrm{sign}(\nabla_{x}L)$
6:▹ Create adversarial example7: $x_{adv}\leftarrow x+\delta$8: **return** 
$x_{adv}$

Projected Gradient Descent (PGD). PGD [17] is an iterative variant that performs multiple gradient steps with projection:

(8) $$x_{adv}^{\left(t+1\right)}=\Pi_{x+S}\left(x_{adv}^{\left(t\right)}+\alpha \cdot \mathrm{sign}\left(\nabla_{x_{adv}^{\left(t\right)}}\ell \left(f\left(x_{adv}^{\left(t\right)}\right),y\right)\right)\right)$$

where $\Pi_{x+S}$ projects onto the $\ell_{\infty }$ ball $S=\{\delta :∥\delta {∥}_{\infty }\leq \epsilon \}$ centered at *x*, α is the step size, and *t* indexes the iteration. We perform *T* iterations, typically with α=ϵ/T. The complete PGD attack algorithm is formalized in Algorithm 2.

**Algorithm 2** Projected Gradient Descent (PGD) attack.**Require:** Model *f*, input $x\in R^{d}$, true label *y*, perturbation budget ϵ, step size α, iterations *T***Ensure:** Adversarial example $x_{adv}$
1: $x_{adv}\leftarrow x$▹ Initialize2: $x_{orig}\leftarrow x$

3: **for** t=1 to *T* **do**4:▹ Compute gradient5:   $L\leftarrow \mathrm{CrossEntropy}(f\left(x_{adv}\right),y)$6:   $\nabla_{x_{adv}}L\leftarrow \frac{\partial L}{\partial x_{adv}}$
7:▹ Update adversarial example8:   $x_{adv}\leftarrow x_{adv}+\alpha \cdot \mathrm{sign}\left(\nabla_{x_{adv}}L\right)$
9:▹ Project to epsilon ball10:   $\delta \leftarrow x_{adv}-x_{orig}$11:   δ←clip(δ,−ϵ,ϵ)12:   $x_{adv}\leftarrow x_{orig}+\delta$13: **end for**14: **return** 
$x_{adv}$

#### 3.2.2. Noise-Based Attacks

Noise-based attacks add stochastic perturbations without requiring model gradients, making them applicable to black-box scenarios and non-differentiable models.

Gaussian Noise Attack. We add i.i.d. Gaussian noise to each feature:

(9) $$x_{adv}=x+\eta ,\;\eta ∼N\left(0,\sigma^{2}I_{d}\right)$$

where σ controls the noise magnitude and $I_{d}$ is the d×d identity matrix.

Uniform Noise Attack. Uniform noise provides bounded perturbations:

(10) $$x_{adv}=x+\eta ,\;\eta ∼U{\left(-\epsilon ,\epsilon \right)}^{d}$$

where each element of η is independently sampled from the uniform distribution.

#### 3.2.3. Feature-Based Attacks

Feature-based attacks exploit the discrete nature of network traffic features through masking and permutation.

Feature Mask Attack. This attack randomly zeros out features to simulate missing or corrupted data:

(11) $$x_{adv}\left[i\right]=\left\{\begin{array}{cc} 0 & \mathrm{if}\;m_{i}=1\\ x[i] & \mathrm{otherwise} \end{array}\right.$$

where $m\in {\left\{0,1\right\}}^{d}$ is a binary mask with $P(m_{i}=1)=r$ for masking ratio r∈[0,1]. The complete procedure is detailed in Algorithm 3.

**Algorithm 3** Feature Mask Attack (Evasion).
**Require:** Input sample $x\in R^{d}$, masking ratio *r*


**Ensure:** Perturbed sample $x_{adv}$
1: $x_{adv}\leftarrow x$
2:▹ Randomly mask features3: M←RandomBinary(d,p=r)▹ Binary mask
4: **for** i=1 to *d* **do**5:   **if** M[i]=1 **then**6:     $x_{adv}\left[i\right]\leftarrow 0$▹ Zero out feature7:   **end if**8: **end for**9: **return** 
$x_{adv}$

Feature Swap Attack. This attack permutes feature values to disrupt learned feature correlations:

(12) $$x_{adv}=\Pi_{I}x$$

where $\Pi_{I}$ represents a partial permutation affecting a fraction *r* of features selected uniformly at random. The implementation details are provided in Algorithm 4.

**Algorithm 4**  Feature Swap Attack.
**Require:** Input sample $x\in R^{d}$, swap ratio *r*
**Ensure:** Perturbed sample $x_{adv}$

  1: $x_{adv}\leftarrow x$  2: $n_{swaps}\leftarrow \left\lfloor d\cdot r\right\rfloor$
  3:▹ Randomly swap feature pairs  4: $I\leftarrow \mathrm{RandomPermutation}(d,2\times n_{swaps})$  5: $I_{1}\leftarrow I\left[1:n_{swaps}\right]$  6: $I_{2}\leftarrow I\left[n_{swaps}+1:2\times n_{swaps}\right]$
  7: **for** j=1 to $n_{swaps}$ **do**  8:   $i_{1}\leftarrow I_{1}\left[j\right]$  9:   $i_{2}\leftarrow I_{2}\left[j\right]$10:   $\mathrm{swap}(x_{adv}\left[i_{1}\right],x_{adv}\left[i_{2}\right])$11: **end for**12: **return** 
$x_{adv}$

#### 3.2.4. Constrained and Adaptive Evaluation

**Figure 2:**
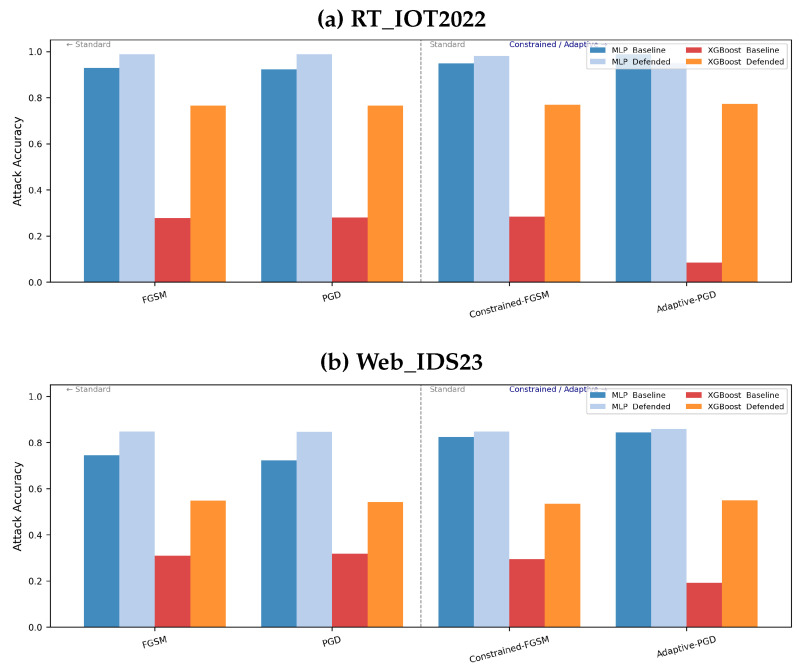
Representative constrained/adaptive attack results for one tree model (XGBoost) and one neural model (Residual MLP). These panels compare standard FGSM/PGD with stronger constrained/adaptive variants.

In the evaluation, we additionally report representative constrained/adaptive attacks for one tree-based model and one neural model. Constrained-FGSM restricts perturbations to valid normalized feature ranges and attacker-controllable coordinates, while Adaptive-PGD targets the defended pipeline end to end, including sanitization, ensemble aggregation, and anomaly-aware confidence adjustment. For neural models, this attack is implemented with a differentiable approximation of the defended path; for tree models, it is implemented through transfer-based or black-box optimization. These results are reported in Figure 2.

### 3.3. Defense Mechanisms

Our defense strategy employs three complementary techniques: input sanitization, ensemble diversity, and anomaly detection.

#### 3.3.1. Input Sanitization

To mitigate adversarial perturbations at the input level, we retain median filtering only as a heuristic feature-squeezing step, not as a semantically grounded local operator for network-flow features. The transformation is therefore evaluated empirically rather than justified by any presumed spatial meaning of adjacent feature indices:

(13) $$x_{clean}\left[i\right]=\mathrm{median}\left\{x\left[i-1\right],x\left[i\right],x\left[i+1\right]\right\}$$

**Figure 3:**
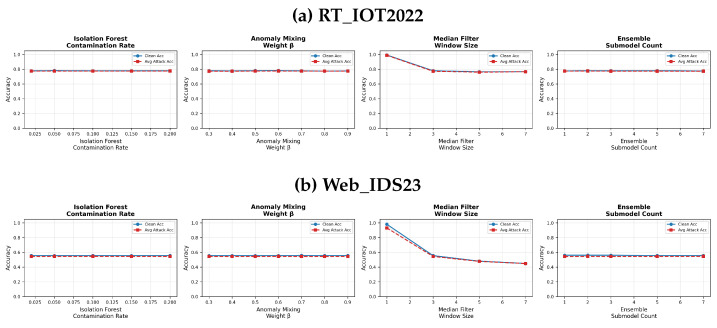
Sensitivity of the defended operating point to contamination rate, anomaly-mixing weight, median-filter window size, and ensemble size.

Unlike image or signal data, IDS feature vectors do not have a canonical neighborhood structure. For that reason, the paper does not claim that median filtering is universally appropriate for tabular NIDS data. Instead, we treat it as an optional outlier-suppression heuristic and explicitly study its window-size sensitivity in Figure 3. Those results show that this component is the most brittle part of the full pipeline and must be tuned cautiously. The detail algorithm is presented in Algorithm 5.

**Algorithm 5**  Input sanitization via median filtering.
**Require:** Input sample $x\in R^{d}$, window size *w*

**Ensure:** Sanitized input $x_{clean}$

1: $x_{clean}\leftarrow x$
2: **for** i=1 to *d* **do**3:▹ Apply median filter to each feature4:  $x_{clean}\left[i\right]\leftarrow \mathrm{median}\left(\left\{x\left[i-1\right],x\left[i\right],x\left[i+1\right]\right\}\right)$▹ Size-3 window5: **end for**6: **return** 
$x_{clean}$

#### 3.3.2. Ensemble Diversity Through Data Augmentation

We train *M* sub-models $\left\{f_{1},f_{2},\ldots ,f_{M}\right\}$ on differently augmented versions of the training data to create a diverse ensemble. For sub-model *m*, the training data is augmented as

(14) $${\tilde{x}}_{i}^{\left(m\right)}=\left\{\begin{array}{cc} x_{i} & \mathrm{if}\;m=1\\ \mathrm{MedianFilter}(x_{i}) & \mathrm{if}\;m=2\\ x_{i}+\eta_{m},\;\eta_{m}∼N\left(0,{\left(0.02m\right)}^{2}I_{d}\right) & \mathrm{if}\;m\geq 3 \end{array}\right.$$

This diversity ensures that different sub-models develop different decision boundaries, making it harder for an adversary to fool all models simultaneously.

#### 3.3.3. Adversarial Training

Following the augmentation phase, we generate pseudo-adversarial examples using multiple attack types:

(15) $$D_{adv}=\left\{\left(x_{i}^{\left(k\right)},y_{i}\right):x_{i}^{\left(k\right)}=A_{k}\left(x_{i}\right),k\in \left\{1,\ldots ,K\right\}\right\}$$

where $A_{k}$ represents the *k*-th attack type. The combined training set becomes

(16) $$D_{combined}=D\cup D_{adv}$$

Each sub-model $f_{m}$ is then retrained on $D_{combined}$ with additional noise injection:

(17) $$\min_{\theta_{m}}\sum_{\left(x,y\right)\in D_{combined}}\ell \left(f_{m}\left(x+\eta_{m};\theta_{m}\right),y\right)$$

where $\theta_{m}$ are the model parameters and $\eta_{m}∼N\left(0,{\left(0.01m\right)}^{2}I_{d}\right)$.

#### 3.3.4. Anomaly Detection

We employ Isolation Forest [50] as an anomaly detector A to identify potential adversarial samples during inference. Isolation Forest assigns an anomaly score based on the path length required to isolate a sample in a random forest:

(18) $$s\left(x\right)=2^{-\frac{E[h(x)]}{c(n)}}$$

where h(x) is the path length, E[·] denotes expectation over trees, and c(n) is a normalization factor. Samples with s(x) above a threshold (corresponding to contamination rate ρ) are flagged as anomalies.

### 3.4. Ensemble Prediction with Anomaly Awareness

During inference, our framework combines predictions from all sub-models while incorporating anomaly detection signals. The complete inference procedure is presented in Algorithm 6, which integrates input sanitization, anomaly detection, and ensemble aggregation into a unified robust prediction mechanism.

**Algorithm 6** Defended ensemble prediction with anomaly detection.
**Require:** Test sample $x\in R^{d}$, Ensemble E={M,A}
**Ensure:** Robust prediction $\hat{y}$ and confidence *c*

  1: **Step 1: Input Sanitization**  2: $x_{clean}\leftarrow \mathrm{MedianFilter}\left(x\right)$
  3: **Step 2: Anomaly Detection**  4: $\mathrm{isAnomaly}\leftarrow A.\mathrm{predict}(x_{clean})=-1$
  5: **Step 3: Multi-Model Prediction**  6: P←∅▹ Probability predictions  7: **for** 
$f_{m}\in M$
 **do**  8:   $p_{m}\leftarrow f_{m}.\text{predict\_proba}\left(x_{clean}\right)$▹ Get probability distribution  9: $P\leftarrow P\cup \{p_{m}\}$10: **end for**
11: **Step 4: Ensemble Aggregation**12: $p_{ensemble}\leftarrow \frac{1}{\left|M\right|}\sum_{p_{m}\in P}p_{m}$▹ Average probabilities
13: **Step 5: Anomaly-Aware Adjustment**14: **if** 
isAnomaly=True
 **then**15:   $p_{uniform}\leftarrow \frac{1}{C}\cdot 1_{C}$▹ Uniform distribution16:   $p_{ensemble}\leftarrow 0.6\cdot p_{ensemble}+0.4\cdot p_{uniform}$17: **end if**
18: **Step 6: Final Prediction**19: $\hat{y}\leftarrow \arg\max_{c}p_{ensemble}\left[c\right]$20: $c\leftarrow \max_{c}p_{ensemble}\left[c\right]$21: **return** 
$\hat{y},c$

#### 3.4.1. Probability Aggregation

Given a test sample *x*, each sub-model $f_{m}$ produces a probability distribution over classes:

(19) $$p_{m}\left(x\right)=\left[p_{m}^{\left(1\right)}\left(x\right),p_{m}^{\left(2\right)}\left(x\right),\ldots ,p_{m}^{\left(C\right)}\left(x\right)\right]$$

where $p_{m}^{\left(c\right)}\left(x\right)=P\left(y=c|x;f_{m}\right)$. The ensemble probability is computed via simple averaging:

(20) $$p_{ensemble}\left(x\right)=\frac{1}{M}\sum_{m=1}^{M}p_{m}\left(x_{clean}\right)$$

where $x_{clean}=\mathrm{MedianFilter}\left(x\right)$.

#### 3.4.2. Anomaly-Aware Adjustment

If the anomaly detector flags $x_{clean}$ as anomalous ($A(x_{clean})=-1$), we apply a conservative adjustment:

(21) $${\tilde{p}}_{ensemble}\left(x\right)=\beta \cdot p_{ensemble}\left(x\right)+\left(1-\beta \right)\cdot p_{uniform}$$

where $p_{uniform}=\left[1/C,1/C,\ldots ,1/C\right]$ is the uniform distribution and β=0.6 controls the mixing weight. This adjustment reduces overconfident predictions on potentially adversarial or out-of-distribution samples.

The value β=0.6 was selected empirically via grid search on the validation set, balancing between preserving the ensemble’s discriminating information (weighted by β) and reducing overconfident predictions on anomalous samples (weighted by 1-β). Values below 0.5 caused excessive uncertainty on clean samples, while values above 0.8 provided insufficient correction on adversarial ones.

#### 3.4.3. Final Prediction

The final class prediction and confidence score are obtained as follows:

(22) $$\hat{y}=\arg\max_{c\in \{1,\ldots ,C\}}{\tilde{p}}_{ensemble}^{\left(c\right)}\left(x\right)$$

(23) $$\mathrm{confidence}=\max_{c\in \{1,\ldots ,C\}}{\tilde{p}}_{ensemble}^{\left(c\right)}\left(x\right)$$

The complete end-to-end inference procedure, integrating all the components described above, is formally presented in Algorithm 6. This algorithm demonstrates how input sanitization, anomaly detection, ensemble aggregation, and anomaly-aware adjustment work together to produce robust predictions.

### 3.5. Defended Evaluation Performance Metrics

To comprehensively assess the effectiveness of our proposed adversarial defense framework, we employ four key evaluation metrics that capture different aspects of model robustness. These metrics evaluate the classification accuracy under various attack scenarios, the success rate of adversarial attacks, the stability of prediction confidence, and the overall improvement achieved by defense mechanisms compared to baseline models.

#### 3.5.1. Accuracy

Accuracy measures the proportion of correctly classified samples and serves as the primary performance indicator for intrusion detection systems. We evaluate accuracy in two settings:

Clean Accuracy. This measures the model’s performance on unperturbed test data and serves as the baseline for comparison:

(24) $${\mathrm{Acc}}_{\mathrm{clean}}=\frac{1}{N_{test}}\sum_{i=1}^{N_{test}}\left[f\left(x_{i}\right)=y_{i}\right]$$

where $N_{test}$ is the number of test samples, $f(x_{i})$ is the predicted label, and $y_{i}$ is the true label. Clean accuracy establishes the upper bound of performance and helps quantify the accuracy sacrifice (if any) introduced by defense mechanisms.

Attack Accuracy. This measures the model’s performance on adversarially perturbed samples. For a given attack type A, it is computed as

(25) $${\mathrm{Acc}}_{A}=\frac{1}{N_{test}}\sum_{i=1}^{N_{test}}\left[f\left(A\left(x_{i}\right)\right)=y_{i}\right]$$

where $A(x_{i})$ denotes the adversarially perturbed version of sample $x_{i}$. Higher attack accuracy indicates stronger robustness against the specific attack type. We evaluate attack accuracy across six different attack types: Gaussian noise, Uniform noise, Feature-Mask, Feature-Swap, FGSM, and PGD.

The average attack accuracy across all attack types provides a comprehensive measure of overall robustness:

(26) $${\mathrm{Acc}}_{\mathrm{attack}}^{\mathrm{avg}}=\frac{1}{\left|A\right|}\sum_{A\in \{\mathrm{attacks}\}}{\mathrm{Acc}}_{A}$$

where |A|=6 denotes the total number of attack types considered in our evaluation.

The robustness gap, defined as the difference between clean and average attack accuracy, quantifies the model’s vulnerability:

(27) $$\mathrm{Gap}={\mathrm{Acc}}_{\mathrm{clean}}-{\mathrm{Acc}}_{\mathrm{attack}}^{\mathrm{avg}}$$

A smaller gap indicates better adversarial robustness, with Gap = 0 representing ideal robustness where the model maintains clean accuracy even under attack.

#### 3.5.2. ASR

ASR quantifies the effectiveness of adversarial attacks by measuring the proportion of originally correctly classified samples that are misclassified after perturbation. This metric provides a direct measure of the adversary’s success in fooling the model. For attack type A, ASR is defined as

(28) $${\mathrm{ASR}}_{A}=\frac{\sum_{i=1}^{N_{test}}\left[f\left(x_{i}\right)=y_{i}\wedge f\left(A\left(x_{i}\right)\right)\neq y_{i}\right]}{\sum_{i=1}^{N_{test}}\left[f\left(x_{i}\right)=y_{i}\right]}$$

where the denominator restricts attention to samples correctly classified under clean conditions, ensuring ASR measures the adversary’s success rate relative to the model’s effective operating set rather than the full test set.

The ASR ranges from 0 to 1, where: ASR = 0 indicates perfect robustness (no successful attacks); ASR = 1 indicates complete vulnerability (all clean predictions are flipped); and lower ASR values represent better defense effectiveness.

ASR provides a complementary perspective to attack accuracy by focusing specifically on the adversary’s success in causing misclassification. While attack accuracy measures overall performance, ASR isolates the impact of attacks on samples that were originally classified correctly, which is particularly relevant when evaluating defense mechanisms designed to prevent prediction flips.

For a comprehensive assessment, we compute the ASR for each of the six attack types and report the average:

(29) $${\mathrm{ASR}}_{\mathrm{avg}}=\frac{1}{\left|A\right|}\sum_{A\in \{\mathrm{attacks}\}}{\mathrm{ASR}}_{A}$$

#### 3.5.3. Confidence Drop

Beyond accuracy metrics, we analyze the confidence scores of model predictions to understand how adversarial perturbations affect prediction certainty. Confidence analysis reveals whether models become uncertain under attack even when predictions remain correct, or conversely, whether they maintain overconfidence on misclassified adversarial examples.

For a sample *x* with predicted probability distribution $p\left(x\right)=[p_{1}\left(x\right),\ldots ,p_{C}\left(x\right)]$, the confidence score is defined as the maximum predicted probability:

(30) $$\mathrm{Confidence}\left(x\right)=\max_{c\in \{1,\ldots ,C\}}p_{c}\left(x\right)$$

The confidence drop under attack A measures the average decrease in predic- tion confidence:

(31) $$\Delta {\mathrm{Confidence}}_{A}=\frac{1}{N_{test}}\sum_{i=1}^{N_{test}}\left[\mathrm{Confidence}\left(x_{i}\right)-\mathrm{Confidence}\left(A\left(x_{i}\right)\right)\right]$$

A large positive confidence drop indicates that adversarial perturbations significantly reduce model certainty, revealing vulnerability even when predictions remain correct. Conversely, a small or negative confidence drop suggests that the model maintains or even increases confidence under attack, which may indicate either robustness or problematic overconfidence on adversarial examples.

For defended models, we expect: smaller confidence drops compared to baseline models, indicating maintained certainty; more calibrated confidence scores where confidence aligns with actual correctness; and reduced overconfidence on misclassified adversarial examples.

The average confidence drop across all attack types provides an overall measure of confidence stability:

(32) $$\Delta {\mathrm{Confidence}}_{\mathrm{avg}}=\frac{1}{\left|A\right|}\sum_{A\in \{\mathrm{attacks}\}}\Delta {\mathrm{Confidence}}_{A}$$

This metric is particularly valuable for understanding the behavioral changes in model predictions under adversarial conditions, complementing the binary correctness information provided by accuracy metrics.

#### 3.5.4. Defense Improvement

Defense improvement measures the relative enhancement in robustness achieved by applying defense mechanisms compared to baseline models without defense. This metric directly quantifies the benefit of our proposed adversarial defense framework and enables comparison across different model architectures and attack scenarios.

For a given attack type A, we compute defense improvement based on the accuracy change from baseline to defended models:

(33) $${\mathrm{DefenseImprovement}}_{A}=\frac{{\mathrm{Acc}}_{A}^{\mathrm{defended}}-{\mathrm{Acc}}_{A}^{\mathrm{baseline}}}{{\mathrm{Acc}}_{A}^{\mathrm{baseline}}}\times 100\%$$

where ${\mathrm{Acc}}_{A}^{\mathrm{defended}}$ and ${\mathrm{Acc}}_{A}^{\mathrm{baseline}}$ represent the attack accuracy of defended and baseline models, respectively.

Alternatively, defense improvement can be measured through robustness gap reduction:

(34) $${\mathrm{DefenseImprovement}}_{\mathrm{gap}}=\frac{{\mathrm{Gap}}^{\mathrm{baseline}}-{\mathrm{Gap}}^{\mathrm{defended}}}{{\mathrm{Gap}}^{\mathrm{baseline}}}\times 100\%$$

where ${\mathrm{Gap}}^{\mathrm{baseline}}$ and ${\mathrm{Gap}}^{\mathrm{defended}}$ are the robustness gaps (clean accuracy minus attack accuracy) for baseline and defended models, respectively.

The interpretation of defense improvement values is as follows: positive values indicate successful defense (improved robustness); values near 100% indicate near-complete gap closure; negative values indicate that the defense mechanism degraded robustness; and zero indicates no improvement from defense.

The average defense improvement across all attacks provides an overall assessment of defense effectiveness:

(35) $${\mathrm{DefenseImprovement}}_{\mathrm{avg}}=\frac{1}{\left|A\right|}\sum_{A\in \{\mathrm{attacks}\}}{\mathrm{DefenseImprovement}}_{A}$$

This metric is particularly valuable for: comparing different defense strategies across models; identifying which attack types are most effectively mitigated; understanding the trade-off between clean accuracy sacrifice and robustness gain; and determining the practical value of deploying defense mechanisms.

#### 3.5.5. Comprehensive Evaluation Framework

These four metrics collectively provide a multi-faceted view of adversarial robustness: Accuracy quantifies correctness on both clean and adversarial data. ASR measures the adversary’s success in flipping predictions. Confidence drop reveals changes in prediction certainty under attack. Defense improvement quantifies the benefit of defense mechanisms.

Table 1 summarizes these evaluation metrics with their mathematical definitions and desired properties.

By examining these complementary metrics across multiple attack types and model architectures, we obtain a comprehensive understanding of the strengths, weaknesses, and trade-offs associated with different adversarial defense strategies for network intrusion detection systems. This multi-metric evaluation ensures that improvements in one dimension (e.g., attack accuracy) are not achieved at unacceptable costs in other dimensions (e.g., clean accuracy or computational efficiency).

## 4. Experiments

This section presents the comprehensive experimental evaluation of our proposed adversarial-robust network intrusion detection framework. We begin by describing the experimental setup and computing infrastructure, followed by detailed descriptions of the datasets used. Finally, we present the hyperparameter configurations for all models and defense mechanisms.

### 4.1. Experimental Setting

All experiments were conducted on a workstation running Windows 10 with an Intel Core i7-10700K CPU (3.80 GHz), 64 GB RAM (Intel, Santa Clara, CA, USA), and the software stack used in the original submission. TensorFlow v. 2.18.0 was used for neural models, while scikit-learn, XGBoost [51], and LightGBM [52] were used for preprocessing and tree-based baselines.

The protocol emphasizes comparative robustness rather than a single headline score. In addition to clean accuracy and per-attack accuracy, we report ASR profiles, defense-improvement trends, representative constrained/adaptive attacks, component-wise ablations against simpler baselines, parameter sensitivity, sample-fraction effects, per-class F1 behavior, repeated-run significance maps, and computational overhead. The significance plots summarize repeated baseline-versus-defended comparisons across multiple runs, and the overhead plots separate training cost from inference cost.

**Figure 4:**
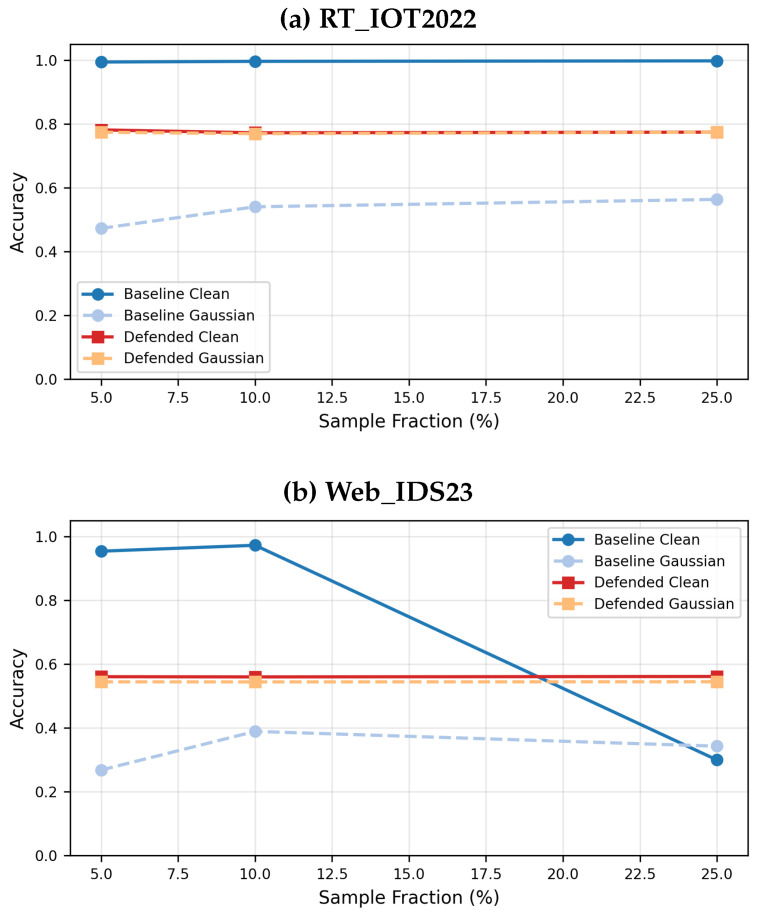
Effect of increasing the dataset sample fraction from 5% to 10% and 25%.

For all datasets, we retain the original stratified train/validation/test split for comparability with the submitted version, and all features are standardized using z-score normalization. However, we now state this limitation explicitly: the evidence is benchmark limited and does not constitute a deployment-complete chronology-aware or source-aware evaluation. To assess how strongly the sampling decision affects the conclusions, we additionally report results at 5%, 10%, and 25% sample fractions (Figure 4).

Standard experiments cover Gaussian, Uniform, Feature-Mask, Feature-Swap, FGSM, and PGD attacks. Representative constrained/adaptive results are reported for one tree model and one neural model (Figure 2) because these two cases expose the two distinct attack interfaces considered in this study: transfer/black-box attacks for trees and direct gradient-based attacks for differentiable networks.

### 4.2. Dataset Description

We evaluate the framework on two benchmark NIDS datasets with different feature structures and attack surfaces: RT_IOT2022 and Web_IDS23. RT_IOT2022 contains IoT-oriented network-flow data with 83 numeric features and a mix of benign traffic and attack classes derived from smart-home and industrial settings. Web_IDS23 contains 85 features derived from HTTP/HTTPS traffic and web-application attack scenarios such as SQL injection, XSS, and related request/response manipulations.

The original submission relied on a 5% stratified sample because of computational cost. In the paper, we preserve that 5% setting for the main headline comparisons so that the baseline and defended results remain directly comparable, but we also add a sample-fraction stability study at 10% and 25% (Figure 4). This allows us to narrow our claims appropriately: RT_IOT2022 appears comparatively stable under larger fractions, whereas Web_IDS23 is more sampling sensitive and therefore requires more cautious interpretation.

Because both datasets remain benchmark testbeds rather than deployment logs, the limits its practical claims to the studied datasets, model families, and perturbation settings. The results should therefore be interpreted as comparative evidence for robust tabular NIDS design, not as a claim of immediate deployment readiness across arbitrary production environments.

### 4.3. Hyperparameter Settings

This section describes the reference hyperparameters used for the main baseline and defended comparisons. Rather than presenting one universal optimum, the treats these values as a reference operating point and studies the sensitivity of the most important defense parameters in Figure 3.

Table 2 summarizes the key hyperparameters for all models used in our experiments.

The values in Table 2 denote the operating point used for the main experiments. In the paper, we additionally vary the Isolation Forest contamination rate, anomaly-mixing weight β, median-filter window size, and ensemble size. The resulting sensitivity curves show that contamination rate, β, and ensemble size are relatively stable around the chosen defaults, whereas the median-filter window is highly sensitive and can dominate the overall clean/robustness trade-off.

## 5. Experimental Results

This results section focuses on seven questions raised by the reviewers: (i) how the defended models behave in absolute clean and attacked accuracy, (ii) whether ASR is reduced, (iii) whether the full four-stage pipeline outperforms simpler alternatives, (iv) whether representative constrained/adaptive attacks change the picture materially, (v) how sensitive the conclusions are to parameter and sample-fraction choices, (vi) what trade-offs emerge in class-wise behavior and computational overhead, and (vii) which effects remain statistically reliable across repeated runs.

### 5.1. Overall Clean and Attack Accuracy

**Figure 5:**
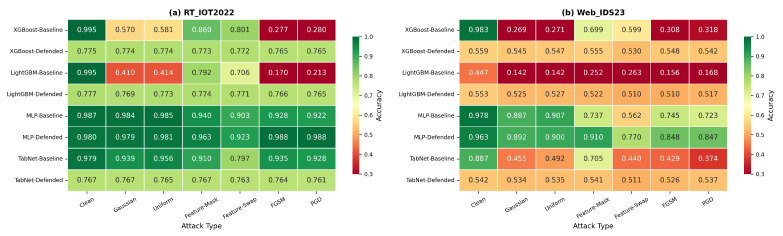
Overall clean and attack accuracy for baseline and defended models on RT_IOT2022 and Web_IDS23. The revised comparison emphasizes absolute performance rather than only relative robustness-gap reduction.

Figure 5 shows that the conclusions are strongly architecture- and dataset dependent. On RT_IOT2022, the largest absolute gains appear for the tree-based models under gradient attacks: XGBoost improves from 0.277/0.280 (FGSM/PGD) to 0.765/0.765, and LightGBM improves from 0.170/0.213 to 0.766/0.765. These gains, however, come with a large clean-accuracy reduction from roughly 0.995 to about 0.776. Residual MLP starts from a much stronger baseline and improves from an average attacked accuracy of about 0.944 to about 0.970 while losing only 0.007 clean accuracy. By contrast, the full four-component defense over-regularizes TabNet on RT_IOT2022, reducing clean accuracy from 0.979 to 0.767 and lowering average attacked accuracy from about 0.911 to about 0.765.

The Web_IDS23 results are even more heterogeneous. XGBoost becomes substantially more uniform across attacks, improving from 0.269–0.318 on Gaussian/Uniform/FGSM/PGD to approximately 0.542–0.548 but only after a severe clean-accuracy drop from 0.983 to 0.559. LightGBM improves both clean and attacked performance, moving from 0.447 clean and 0.187 average attacked accuracy to 0.553 clean and 0.519 attacked accuracy. Residual MLP again offers the most balanced behavior, increasing average attacked accuracy from about 0.760 to about 0.861 with only a small clean decrease from 0.978 to 0.963. TabNet becomes more robust in a gap-reduction sense, but the defended model largely collapses to a uniform band around 0.53 for both clean and attacked inputs.

### 5.2. ASR Profiles and Confidence Stability

**Figure 6:**
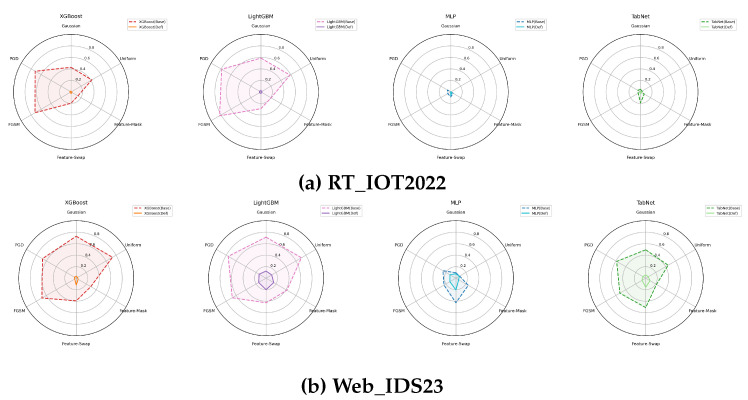
ASR vulnerability profiles for baseline and defended models on both datasets. Smaller defended polygons indicate lower attack success.

The ASR radar plots in Figure 6 confirm that the tree-based models benefit most dramatically from defense on RT_IOT2022: the defended polygons for XGBoost and LightGBM shrink almost to the origin, especially for FGSM and PGD. Residual MLP and TabNet already have comparatively small baseline ASR on RT_IOT2022, so the room for additional reduction is limited. On Web_IDS23, all defended models reduce ASR relative to their baselines, but the reductions are less uniform and do not imply a single universally dominant architecture.

**Figure 7:**
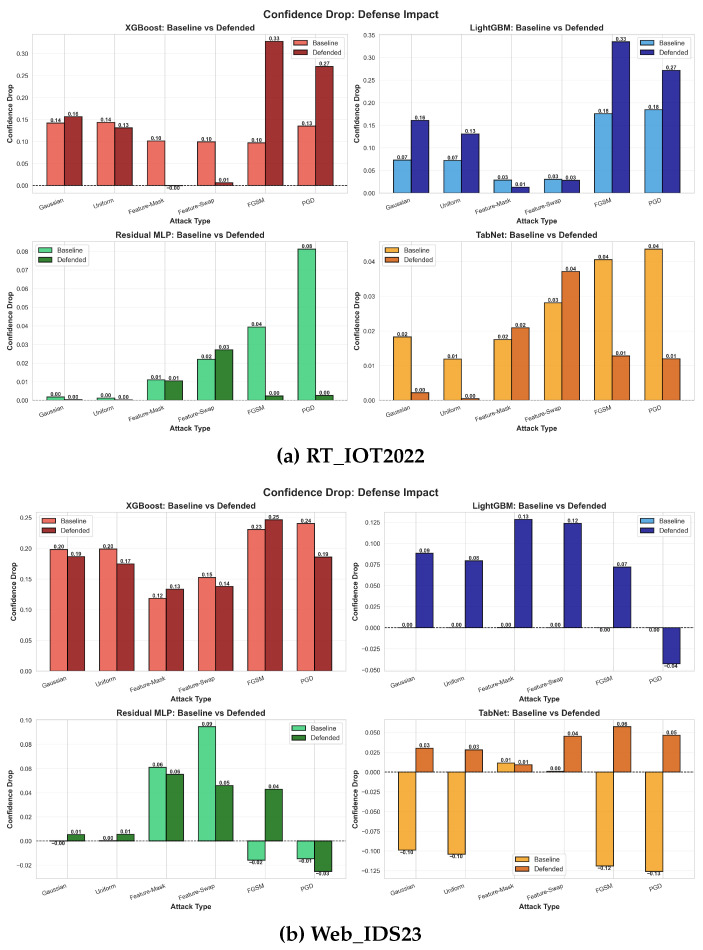
Confidence-drop comparison retained from the original submission. The paper uses confidence stability as a supporting calibration indicator rather than as a primary claim of defense success.

The confidence-drop plots in Figure 7 are consistent with the accuracy trade-off analysis: defended models generally become more conservative under attack, but flatter confidence behavior is not sufficient on its own to justify large clean-accuracy losses. We therefore retain confidence stability as a supporting metric and avoid interpreting calibration improvements as evidence of universal deployment readiness.

### 5.3. Attack-Wise Accuracy and Defense Improvement

**Figure 8:**
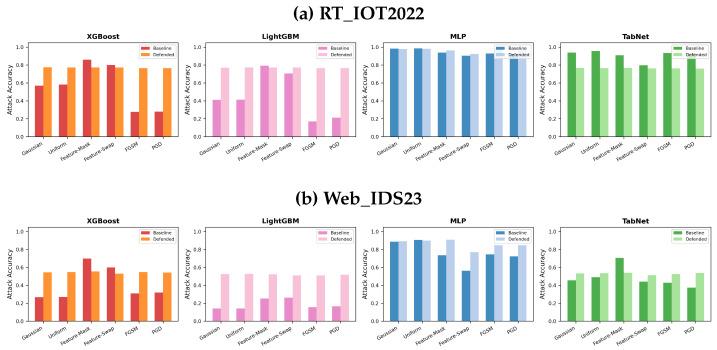
Attack-wise accuracy comparison for baseline and defended models on both datasets.

**Figure 9:**
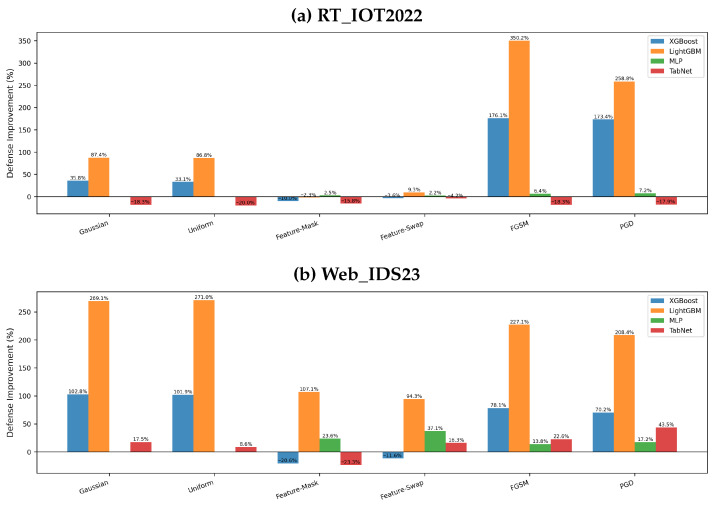
Attack-wise defense improvement measured as relative accuracy change. Positive bars indicate successful defense; negative bars indicate degradation on that attack.

Figure 8 and Figure 9 make two patterns explicit. First, the largest gains for tree models occur on gradient attacks. On RT_IOT2022, LightGBM improves by about 350.2% on FGSM and 258.8% on PGD, while XGBoost improves by 176.1% and 173.4% on the same attacks. Second, these gains are not universal across all components and all datasets. On RT_IOT2022, the full defense degrades TabNet on every attack (roughly −18% to −20% on most attacks), and XGBoost is slightly worse on Feature-Mask and Feature-Swap. On Web_IDS23, XGBoost remains negative on the two feature-based attacks (−20.6% and −11.6%), and TabNet is negative on Feature-Mask (−23.3%) despite improving on noise and gradient attacks.

These attack-wise results are important because they prevent an overly optimistic reading of aggregate robustness metrics. A defense can reduce the overall robustness gap yet still degrade a relevant subset of attacks. In the paper, we therefore treat attack-specific degradation as a primary finding rather than a footnote.

### 5.4. Component-Wise Ablation and Simpler Baselines

**Figure 10:**
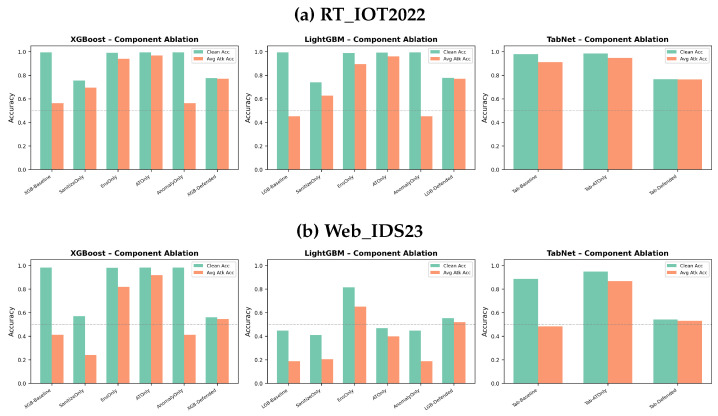
Component-wise ablation against simpler baselines. “SanitizeOnly”, “EnsOnly”, “ATOnly”, and “AnomalyOnly” isolate the main defense components.

Figure 10 directly addresses the reviewers’ concern that the full four-component pipeline may be more complex than necessary. The ablation shows that the full stack rarely dominates the simpler baselines shown here. On RT_IOT2022, adversarial-training-only and ensemble-only are consistently stronger than the full defense for XGBoost and LightGBM, while TabNet-ATOnly clearly outperforms the full TabNet defense. On Web_IDS23, the discrepancy is even larger: XGBoost-ATOnly achieves the best average attacked accuracy (approximately 0.92) while maintaining near-baseline clean accuracy, far above the full defended model (approximately 0.54 attacked accuracy). LightGBM-EnsOnly and TabNet-ATOnly similarly outperform the full stack by wide margins.

The ablation also reveals that median filtering and anomaly-only calibration do not drive the main gains. Sanitization-only is modest at best and often harmful, while anomaly-only is consistently weak. In contrast, adversarial training and ensemble diversity account for most of the useful robustness behavior. This is a central figure outcome: the full pipeline should not be considered the default best configuration simply because it combines more components.

### 5.5. Representative Constrained and Adaptive Attacks

Figure 2 shows that the representative constrained/adaptive evaluations do not uncover a hidden collapse of the defended pipeline. For Residual MLP, defended accuracy stays close to the standard-attack values on both datasets, and the baseline-defended gap remains substantial. For XGBoost, the defended model remains stable around 0.77 on RT_IOT2022 and around 0.54–0.55 on Web_IDS23, whereas the baseline remains highly vulnerable. The adaptive variants therefore reduce performance only modestly relative to the standard attacks in these representative cases.

At the same time, we intentionally limit the scope of this claim. The adaptive figure is reported for representative tree and neural models rather than as an exhaustive all-model benchmark, and the tree-model results should be interpreted under a transfer/black-box threat model rather than as true white-box gradients. This narrower presentation aligns the paper with what the experiments actually support.

### 5.6. Parameter Sensitivity and Sample-Fraction Effects

Figure 3 shows that contamination rate, anomaly-mixing weight, and ensemble size are relatively stable over the examined ranges. In contrast, the median-filter window is highly brittle. On RT_IOT2022, moving from the identity case to larger windows immediately drops accuracy from approximately 0.99 to about 0.78, and on Web_IDS23 the same change reduces clean accuracy from about 0.98 to the 0.45–0.56 range. This is the clearest empirical evidence in the figure that median filtering should be treated as an optional heuristic rather than as a universally justified tabular-defense primitive.

Figure 4 narrows our claims about data protocol. RT_IOT2022 is relatively stable across 5%, 10%, and 25% fractions, with the defended Gaussian accuracy staying close to 0.77 and the baseline clean accuracy remaining near perfect. Web_IDS23 is more sensitive: the baseline clean accuracy changes markedly across fractions, whereas the defended clean and Gaussian performance remains close to 0.55. The paper therefore keeps the original 5% setting for comparability but explicitly treats the Web_IDS23 conclusions as more sample sensitive.

### 5.7. Clean/Robustness Trade-Off and Computational Overhead

**Figure 11:**
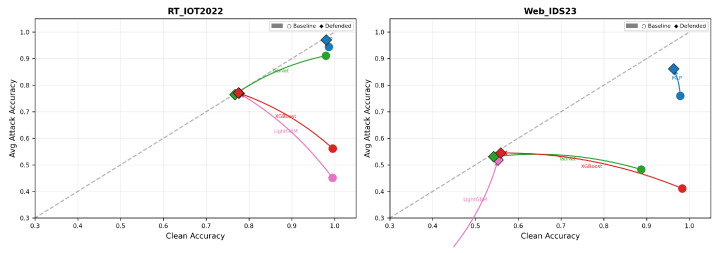
Clean-accuracy versus average attacked-accuracy trade-off. Points above and closer to the diagonal indicate better balance between clean and adversarial performance.

Figure 11 shows why robustness-gap reduction must be interpreted jointly with absolute accuracy. On RT_IOT2022, XGBoost and LightGBM move from high-clean/low-robustness to roughly balanced clean/robustness after defense, but only by sacrificing about 0.22 clean accuracy. Residual MLP, in contrast, moves upward with minimal leftward shift and therefore offers the most favorable Pareto behavior. TabNet is a cautionary case: the defended point lies on a much lower diagonal band than the baseline, meaning the small robustness gap is achieved mainly by lowering both clean and attacked accuracy. On Web_IDS23, XGBoost and TabNet again trade away large amounts of clean accuracy to obtain more uniform attacked performance, while MLP and LightGBM show more useful upward movement.

**Figure 12:**
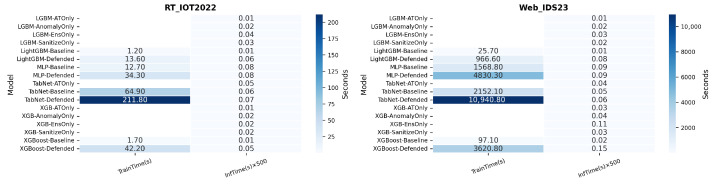
Training and inference overhead for baseline, defended, and representative ablation variants.

The overhead heatmap in Figure 12 shows that the computational cost of the full defense is dominated by retraining rather than inference. Inference overhead remains modest (roughly 0.05–0.15 s in the reported scaling), but training time increases sharply, especially on Web_IDS23: defended TabNet requires about 10,940.8 s, defended MLP about 4830.3 s, and defended XGBoost about 3620.8 s. These results reinforce the ablation finding that simpler baselines should be preferred when they deliver comparable robustness at much lower cost.

### 5.8. Per-Class Behavior and Repeated-Run Significance

**Figure 13:**
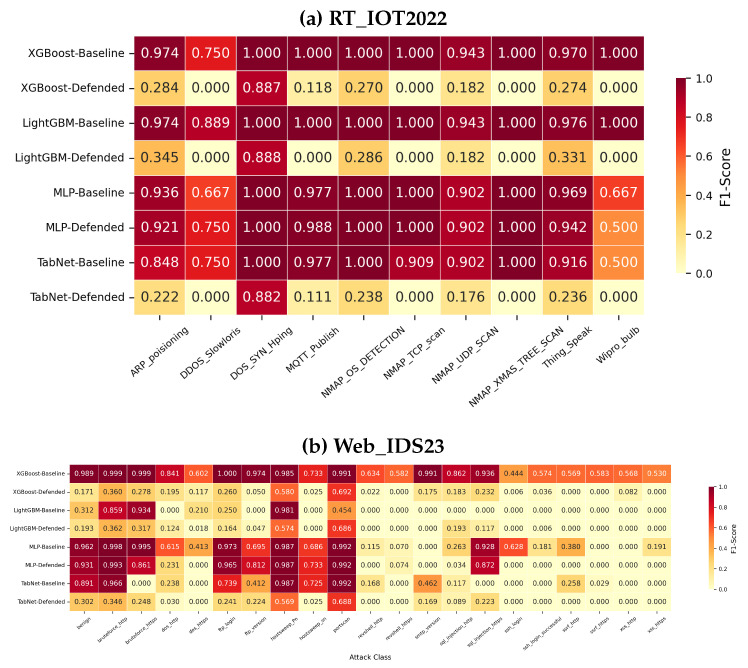
Per-class F1-score on clean data for baseline and defended models.

Figure 13 shows that overall accuracy can hide severe class-wise failures. On RT_IOT2022, the defended tree models and defended TabNet collapse several classes to near-zero F1, while defended Residual MLP largely preserves the original class-wise structure. On Web_IDS23, many low-frequency classes remain difficult for all models, and the defended variants often trade aggregate robustness for severe degradation on minority classes such as revshell, ssh, and several XSS-related labels. This class-wise view is one reason the paper avoids treating a small aggregate gap as sufficient evidence of practical deployability.

**Figure 14:**
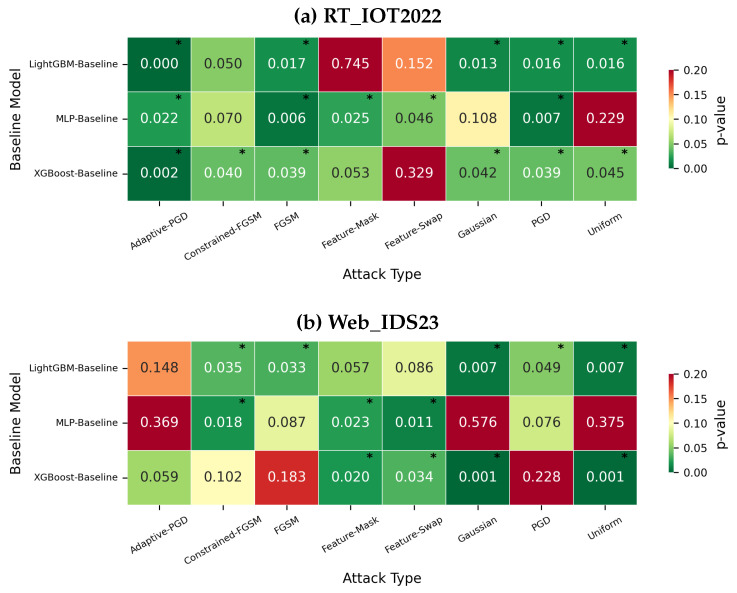
Repeated-run *p*-values for representative baseline-versus-defended attack comparisons. Cells marked with an asterisk indicate *p* < 0.05.

Figure 14 adds the requested statistical caution. On RT_IOT2022, many improvements for XGBoost and LightGBM are statistically significant for Gaussian, Uniform, PGD, and several constrained/adaptive comparisons, whereas Feature-Swap is not consistently significant. On Web_IDS23, significance is mixed: constrained-FGSM and some feature/noise attacks remain significant for XGBoost and LightGBM, but adaptive-PGD and PGD are not uniformly significant across models. The paper therefore reserves strong ranking language for the most stable patterns only.

### 5.9. Cross-Dataset Transfer of the Robustness Gap

**Figure 15:**
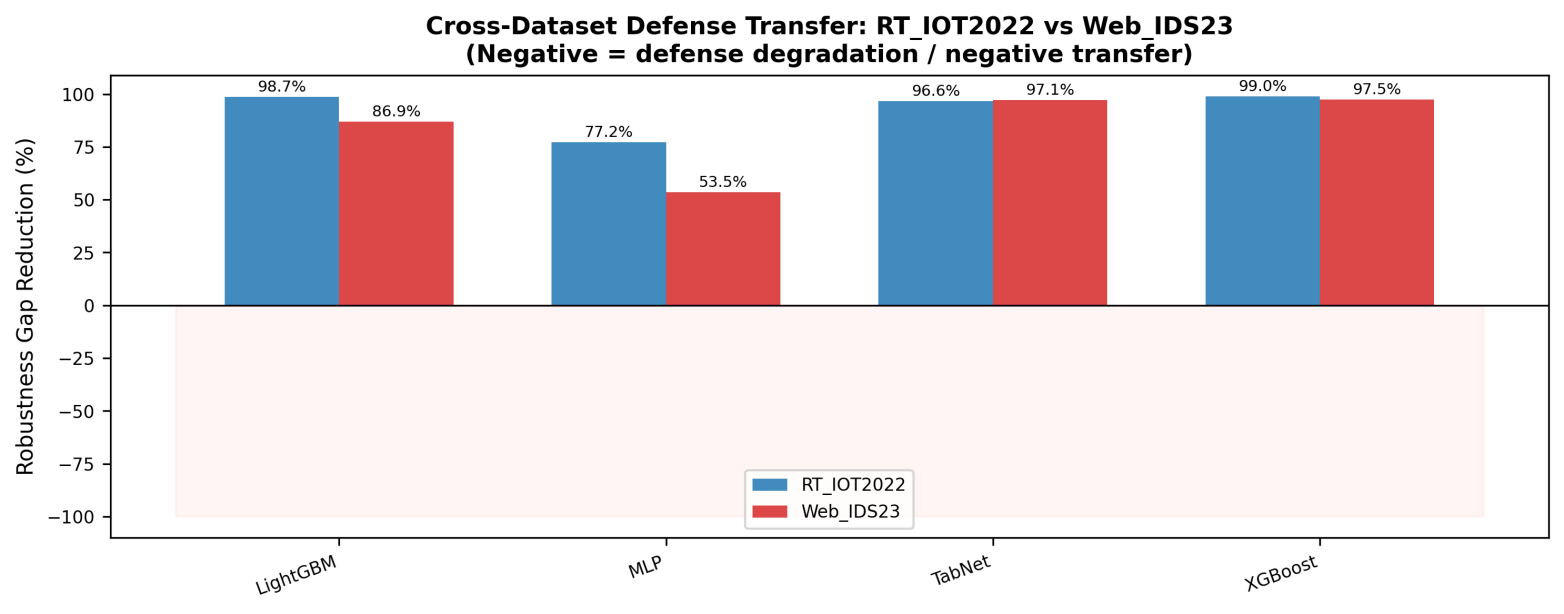
Cross-dataset robustness-gap reduction. Positive values indicate that the defended model closes part of the clean-versus-attack gap; lower values on Web_IDS23 indicate weaker transfer of the defense benefit.

Figure 15 clarifies an important point from the figure: aggregate robustness-gap reduction remains positive on both datasets for all four model families, but the magnitude of that reduction attenuates on Web_IDS23 for every model. The drop is especially pronounced for Residual MLP (77.7% to 53.3%) and LightGBM (98.7% to 86.7%). However, this figure must be read together with Figure 5 and Figure 11, because a small defended gap can also arise when clean accuracy collapses together with attack accuracy. The paper therefore frames the central finding as dataset-dependent transfer attenuation and attack-specific degradation, not as blanket proof of either universal success or universal failure.

## 6. Discussion

The experiments change the interpretation of the paper in three important ways. First, the full four-component defense should not be viewed as a universal best practice. The ablation results show that adversarial training and ensemble diversity explain most of the useful robustness behavior, while median filtering and anomaly-only calibration contribute relatively little and can even be harmful. Second, aggregate robustness-gap reduction is not sufficient on its own. A model can appear “robust” by shrinking the gap through clean-accuracy collapse as demonstrated most clearly by the defended TabNet configurations. Third, robustness claims for tabular NIDS models are strongly dataset dependent even when the same pipeline and hyperparameters are reused.

### 6.1. Updated Performance Summary

Table 3 makes the central trade-off explicit. For XGBoost and LightGBM on RT_IOT2022, the gap is almost completely closed but only after a large drop in clean accuracy. Residual MLP shows a smaller nominal gap reduction, yet it achieves the best absolute balance because both clean and attacked accuracy remain high. TabNet illustrates why gap reduction cannot be interpreted in isolation: the defended gap is tiny, but it is small largely because the defended model predicts at roughly the same moderate level on both clean and attacked inputs.

### 6.2. What in Practical Terms

From a deployment perspective, the evidence suggests three practical rules. First, use simpler baselines as the default benchmark before adopting the full stack. In our experiments, adversarial-training-only or ensemble-only variants often dominate the full defense in both accuracy and cost. Second, treat median filtering as optional and highly data dependent. The sensitivity curves show that larger windows can destroy performance, especially on Web_IDS23. Third, do not rely on a single scalar robustness metric. Absolute clean accuracy, per-attack accuracy, per-class behavior, significance, and overhead must be considered jointly.

These points also change the interpretation of cross-dataset transfer. The paper does not claim that the defended pipeline generalizes uniformly across environments. Instead, it shows that transfer is heterogeneous: some models keep a large gap reduction on both datasets, but the absolute clean/robust balance, minority-class behavior, and computational cost can still differ sharply.

## 7. Conclusions

This paper presents a comparative robustness evaluation of a multi-component defense pipeline for tabular network intrusion detection rather than a claim of a universal new defense primitive. Across XGBoost, LightGBM, TabNet, and Residual MLP, the experiments show that defense behaviour is strongly dependent on model family, dataset, attack type, and evaluation metric. Tree-based models can close most of the clean-versus-attack gap on RT_IOT2022 but often at a large clean-accuracy cost. Residual MLP offers the most favorable overall trade-off in the experiments, while the full four-component pipeline can over-regularize TabNet.

Simpler alternatives, such as adversarial-training-only and ensemble-only, frequently outperform the full defense stack, median filtering is the most brittle component; representative constrained/adaptive attacks do not erase the main robustness patterns. Training overhead can become substantial on the larger Web_IDS23 setting. Most importantly, aggregate gap reduction must be considered in conjunction with absolute clean and accurate classification, per-class behavior, and computational cost.

We therefore conclude with a narrower and more operationally useful claim: robust tabular NIDS defense is validation driven and dataset specific. The full defense stack should not be deployed as a universal default without dataset-specific ablation, sensitivity analysis, and cost–benefit validation on the target environment.

## Figures and Tables

**Table 1:** Summary of evaluation metrics.

Metric	Mathematical Definition	Desired Property
Clean Accuracy	$\frac{1}{N}\sum_{i=1}^{N}\left[f\left(x_{i}\right)=y_{i}\right]$	Higher is better
Attack Accuracy	$\frac{1}{N}\sum_{i=1}^{N}\left[f\left(A\left(x_{i}\right)\right)=y_{i}\right]$	Higher is better
ASR	$\frac{\#\mathrm{successful}\mathrm{attacks}}{\#\mathrm{correct}\mathrm{clean}\mathrm{predictions}}$	Lower is better (0 = perfect defense)
Confidence Drop	Avg[Conf(x)−Conf(A(x))]	Lower is better (stable confidence)
Defense Improvement	$\frac{{\mathrm{Gap}}^{\mathrm{base}}-{\mathrm{Gap}}^{\mathrm{def}}}{{\mathrm{Gap}}^{\mathrm{base}}}\times 100\%$	Higher is better (positive values)

**Table 2:** Summary of key hyperparameters.

Model	Parameter	Value
XGBoost/LightGBM	Number of Estimators	500
	Maximum Depth	8
	Learning Rate	0.1
	Number of Sub-models	5
TabNet	Decision/Attention Dim ($n_{d}$, $n_{a}$)	64
	Decision Steps	5
	Gamma (γ)	1.5
	Learning Rate	0.02
	Batch Size	256
	Max Epochs	100
Residual MLP	Hidden Dimensions	[256, 128, 64]
	Dropout Rate	0.3, 0.2
	L2 Regularization	$10^{-4}$
	Learning Rate	$10^{-3}$
	Batch Size	128
Defense	Contamination Rate	0.1
	Median Filter Size	3
	Anomaly Adjustment	0.6
Attacks	FGSM/PGD ϵ	0.15
	PGD Steps	10
	Feature-Mask Ratio	0.3
	Feature-Swap Ratio	0.2
	Gaussian/Uniform Noise	0.15, [−0.2, 0.2]

**Table 3:** Updated clean/robust accuracy summary for baseline and defended models. Average attacked accuracy is computed over Gaussian, Uniform, Feature-Mask, Feature-Swap, FGSM, and PGD.

Dataset	Model	Clean		Avg. Attack		Gap		Gap Red.
		Base	Def.	Base	Def.	Base	Def.	(%)
RT_IOT2022	XGBoost	0.995	0.775	0.562	0.771	0.434	0.005	98.96
RT_IOT2022	LightGBM	0.995	0.777	0.451	0.770	0.544	0.007	98.65
RT_IOT2022	MLP	0.987	0.980	0.944	0.970	0.043	0.010	77.69
RT_IOT2022	TabNet	0.979	0.767	0.911	0.765	0.068	0.003	96.33
Web_IDS23	XGBoost	0.983	0.559	0.411	0.545	0.572	0.015	97.47
Web_IDS23	LightGBM	0.447	0.553	0.187	0.519	0.260	0.035	86.72
Web_IDS23	MLP	0.978	0.963	0.760	0.861	0.218	0.102	53.25
Web_IDS23	TabNet	0.887	0.542	0.483	0.531	0.405	0.011	97.20

## Data Availability

Available upon reader request.
